# ARKOMA dataset: An open-source dataset to develop neural networks-based inverse kinematics model for NAO robot arms

**DOI:** 10.1016/j.dib.2023.109727

**Published:** 2023-10-27

**Authors:** Arif Nugroho, Eko Mulyanto Yuniarno, Mauridhi Hery Purnomo

**Affiliations:** a Department of Electrical Engineering, Institut Teknologi Sepuluh Nopember (ITS), Surabaya, Indonesia; b Department of Computer Engineering, Institut Teknologi Sepuluh Nopember (ITS), Surabaya, Indonesia; c University Center of Excellence on Artificial Intelligence for Healthcare and Society (UCE AIHeS), Indonesia

**Keywords:** NAO robot arms, Robotic motion control, Inverse kinematics, Data-driven modeling technique, Neural networks, Training dataset, Validation dataset, Testing dataset

## Abstract

The inverse kinematics plays a vital role in the planning and execution of robot motions. In the design of robotic motion control for NAO robot arms, it is necessary to find the proper inverse kinematics model. Neural networks are such a data-driven modeling technique that they are so flexible for modeling the inverse kinematics. This inverse kinematics model can be obtained by means of training neural networks with the dataset. This training process cannot be achieved without the presence of the dataset. Therefore, the contribution of this research is to provide the dataset to develop neural networks-based inverse kinematics model for NAO robot arms. The dataset that we created in this paper is named ARKOMA. ARKOMA is an acronym for ARif eKO MAuridhi, all of whom are the creators of this dataset. This dataset contains 10000 input-output data pairs in which the end-effector position and orientation are the input data and a set of joint angular positions are the output data. For further application, this dataset was split into three subsets: training dataset, validation dataset, and testing dataset. From a set of 10000 data, 60 % of data was allocated for the training dataset, 20 % of data for the validation dataset, and the remaining 20 % of data for the testing dataset. The dataset that we provided in this paper can be applied for NAO H25 v3.3 or later.

Specifications Table

**Table:** 

Subject	Electrical and Electronic Engineering, Computer Science, Control and Systems Engineering, and Artificial Intelligence
Specific subject area	Robotics, Computational Intelligence, Machine Learning
Data format	Analyzed, Filtered
Type of data	Tabular data in CSV format
Data collection	The data acquisition was conducted by using the robotic arms of the NAO H25 v3.3. The initial step in this data acquisition is to create 10000 robotic arm poses through choregraphe software. Thereafter, we can extract these robotic arm poses to obtain the data containing a set of joint angular positions. By feeding a set of the joint angular positions into the forward kinematics model, we can then obtain the corresponding end-effector position and orientation in the cartesian space. In short, this data will be eventually divided into the training dataset, validation dataset, and testing dataset. Note that the dataset provided in this paper is compatible with the NAO H25 v3.3 or later due to the fact that they have the same kinematic structure.
Data source location	Institution: Institut Teknologi Sepuluh Nopember City/Town/Region: Surabaya Country: Indonesia
Data accessibility	Repository name: Mendeley Data and IEEE Dataport Data identification number: Mendeley Data: 10.17632/brg4dz8nbb.1 IEEE Dataport: 10.21227/e7zb-nb33 Direct URL to data: https://data.mendeley.com/datasets/brg4dz8nbb/1 https://ieee-dataport.org/documents/arkoma-dataset-build-neural-networks-based-inverse-kinematics-nao-robot-arms
Related research article	A. Nugroho, E. M. Yuniarno, and M. H. Purnomo, “An Improved Performance of the Designed Robotic Motion Control for NAO Robot Arms Using Hybrid Neural Network-Jacobian,” Int. J. Intell. Eng. Syst., vol. 16, no. 5, pp. 523–538, 2023, doi:10.22266/ijies2023.1031.45[1]

## 1 Value of the Data

• In the design of the robotic motion control for NAO robot arms, it is required to obtain the proper inverse kinematics model. Inverse kinematics is such a highly nonlinear problem that it is frequently difficult to solve analytically. This problem can be overcome by neural networks. The dataset provided in this paper can be utilized by NAO robot developers to develop the inverse kinematics model based on neural networks.
• This dataset contains the input-output data pairs, so this is suitably addressed on supervised learning. Supervised learning is a paradigm where the neural networks are trained by using labeled data. Labeled data means that each input has its corresponding output or desired target. In other words, labeled data is the dataset that comprises the input-output data pairs. In supervised learning, there are different neural network architectures, such as multi-layer perceptron neural networks (MLPNN), convolutional neural networks (CNN), radial basis function neural networks (RBFNN), and so on. By using the dataset, NAO robot developers can develop different neural network architectures to obtain the inverse kinematics model.
• This dataset was divided into three subsets: training dataset, validation dataset, and testing dataset. The training dataset is used to train neural networks. The validation dataset is then utilized to validate the performance of neural networks during the training process, while the testing dataset is employed after the training process to test the performance of the trained neural networks. Therefore, the dataset that we provided in this paper can be applied to not only train neural networks but also validate and test the performance of the trained neural networks-based inverse kinematics model for NAO robot arms.

## 2 Objective

Inverse kinematics plays a significant role in various applications related to robot motions. This is because the planning and execution of appropriate robot motions are inexorable from the inverse kinematics role. It is important to note that most tasks to be performed by the robotic arm are defined in the cartesian space. To perform manipulation tasks such as grasping objects, pick-and-place objects, and path tracking, the control inputs are given in terms of the desired position and orientation of the robot end-effector in the cartesian space. The manipulation tasks in industrial manufacturing for the welding robot, painting robot, and assembling robot are also determined in the cartesian space. However, what we can directly control from the robotic arm to perform the given tasks is its joint actuators that operate in the joint space. Therefore, the only way to solve this problem is through the inverse kinematics. The inverse kinematics deals with the mapping from the robot end-effector operational space, which is the cartesian space, to the robot joint space. The inverse kinematics is specifically used to find a set of joint angular positions based on the given position and orientation of the robot end-effector. The joint angular positions obtained from the inverse kinematics are required to control the joint actuators so that the robot end-effector can be driven to the desired targets in the cartesian space [1].

Due to its nonlinearity, solving the inverse kinematics becomes a complex and challenging task. In general, the inverse kinematics can be solved by using analytical and computational methods. The analytical methods highly depend on the robot kinematics structure, so they are applicable to only a certain robot structure. In the case of robotic arms, Pieper's criterion stated that the inverse kinematics can be possibly solved by the analytical methods when the robotic arms contain a spherical wrist whose three revolute joint axes intersect at a common point [2]. Otherwise, solving the inverse kinematics by using the analytical methods becomes difficult to achieve. Unfortunately, the kinematic structure of NAO robot arms does not meet that criterion. The computational methods are more flexible in solving the inverse kinematics due to the fact that they are not dependent on the robot kinematics structure. The metaheuristic algorithms are such computational methods frequently used to solve the inverse kinematics. The metaheuristic algorithms, such as the particle swarm optimization [3], grey wolf optimization [4], and firefly algorithm [5], have been widely applied for solving the inverse kinematics of any robot. They are such stochastic optimization methods that their convergence to the desired solution highly depends on the random initializations. These methods may not find an optimal solution when the initial guess is not appropriate. In this condition, the objective function resulted from the metaheuristic algorithms is vulnerable to being trapped in local minima. As a consequence, the obtained inverse kinematics solution becomes unacceptable. Hence, to overcome the remaining problems of the aforementioned methods, we can use neural networks. Neural networks have the capability to learn from the patterns of the data through a process called training [6]. Their capability to learn from the data makes neural networks more flexible for solving any complex problem than the aforementioned methods.

Neural networks are such a data-driven modeling technique that they require the dataset to create the inverse kinematics model. The presence of the dataset is a fundamental requirement in neural networks. It means that the presence of the dataset becomes a prerequisite in the use of neural networks. For that reason, we are motivated to create the dataset and disseminate this dataset through this paper. The dataset that we provided in this paper can be widely used by the NAO robot developers to build the neural networks-based inverse kinematics model for NAO robot arms. It can be only achievable with the presence of the dataset. Through this dataset, neural networks can learn from the patterns of the data to find the relationship model between the inputs and outputs in the dataset. In this dataset, the inputs are the robot end-effector position and orientation, whereas the outputs are a set of joint angular positions. This relationship model resulted from neural networks is so-called the neural networks-based inverse kinematics model.

**Fig. 1:**
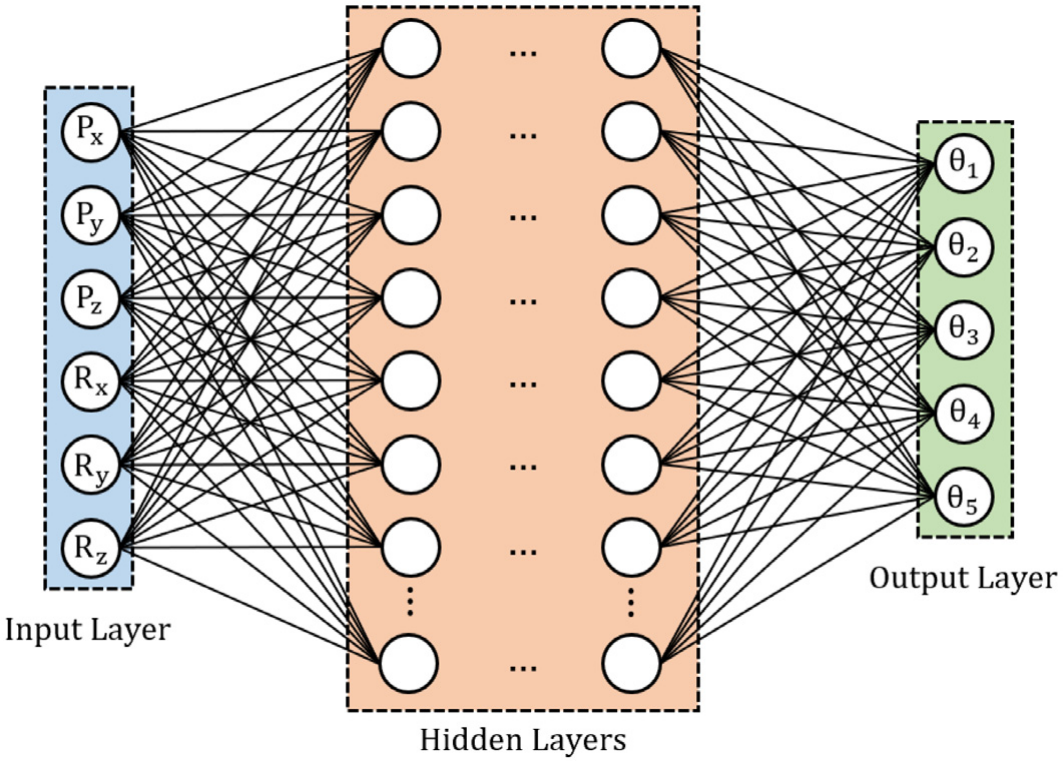
Neural networks-based inverse kinematics model.

**Fig. 2:**
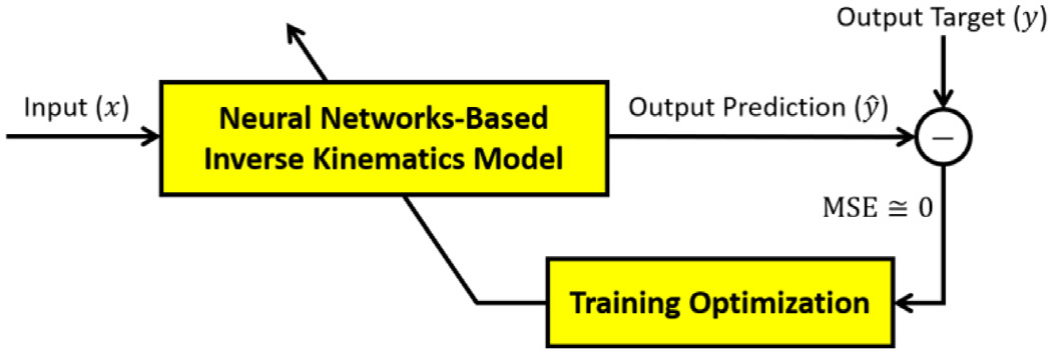
Training process to find the neural networks-based inverse kinematics model.

Based on the number of the input and output parameters in the dataset, the neural networks-based inverse kinematics model will have six neurons in the input layer and five neurons in the output layer, as shown in Fig. 1. In between the input and output layers, there are hidden layers. To determine the number of hidden layers and the neurons in each of them, it is required to train neural networks by feeding the dataset into neural networks [6]. Through the training process, all of us can adjust the neural networks’ hyperparameters to find the optimal number of hidden layers and their neurons. As seen in Fig. 2, the training process must be conducted until reaching a condition where the loss function in terms of mean squared error (MSE) approximates zero.

## 3 Data Description

The dataset that we published in this data repository [7,8] contains input-output data pairs. In this dataset, the input data is the end-effector position and orientation, while the output data is a set of joint angular positions. In this case, the end-effector position and orientation are in the cartesian space, and a set of joint angular positions are in the joint space. For the input data, there are six input parameters where the end-effector position is denoted by the position vectors [$P_{x},P_{y},P_{z}$], and the end-effector orientation is expressed by the rotation vectors [$R_{x},R_{y},R_{z}$]. It is important to note that the end-effector position is referred to as the position of the NAO's hands with respect to the torso, and the end-effector orientation refers to the orientation of the NAO's hands frame relative to the torso's coordinate frame. The torso, which is located in the center of the NAO robot body, is defined as the base coordinate frame. To sum up, the input parameters for the inverse kinematics are entirely described in Table 1.

**Table 1:** The input parameters for the inverse kinematics.

Notation	Description
$P_{x}$	The end-effector position with respect to the torso's x-axis
$P_{y}$	The end-effector position with respect to the torso's y-axis
$P_{z}$	The end-effector position with respect to the torso's z-axis
$R_{x}$	The end-effector orientation relative to the torso's x-axis
$R_{y}$	The end-effector orientation relative to the torso's y-axis
$R_{z}$	The end-effector orientation relative to the torso's z-axis

Meanwhile, for the output data, there are five output parameters. The number of the output parameters for the inverse kinematics is equal to the number of joints. Physically, the NAO robot has five revolute joints for each arm. These revolute joints are the shoulder pitch ($\theta_{1}$), shoulder roll ($\theta_{2}$), elbow yaw ($\theta_{3}$), elbow roll ($\theta_{4}$), and wrist yaw ($\theta_{5}$). These revolute joints have different operational ranges, all of which operate in radians as shown in Table 2 [9].

**Table 2:** The output parameters for the inverse kinematics.

Notation	Left Arm		Right Arm	
	Joint	Range (rad)	Joint	Range (rad)
$\theta_{1}$	LShoulder Pitch	-2.0857 to 2.0857	RShoulder Pitch	-2.0857 to 2.0857
$\theta_{2}$	LShoulder Roll	-0.3142 to 1.3265	RShoulder Roll	-1.3265 to 0.3142
$\theta_{3}$	LElbow Yaw	-2.0857 to 2.0857	RElbow Yaw	-2.0857 to 2.0857
$\theta_{4}$	LElbow Roll	-1.5446 to -0.0349	RElbow Roll	0.0349 to 1.5446
$\theta_{5}$	LWrist Yaw	-1.8238 to 1.8238	RWrist Yaw	-1.8238 to 1.8238

## 4 Experimental Design

**Fig. 3:**
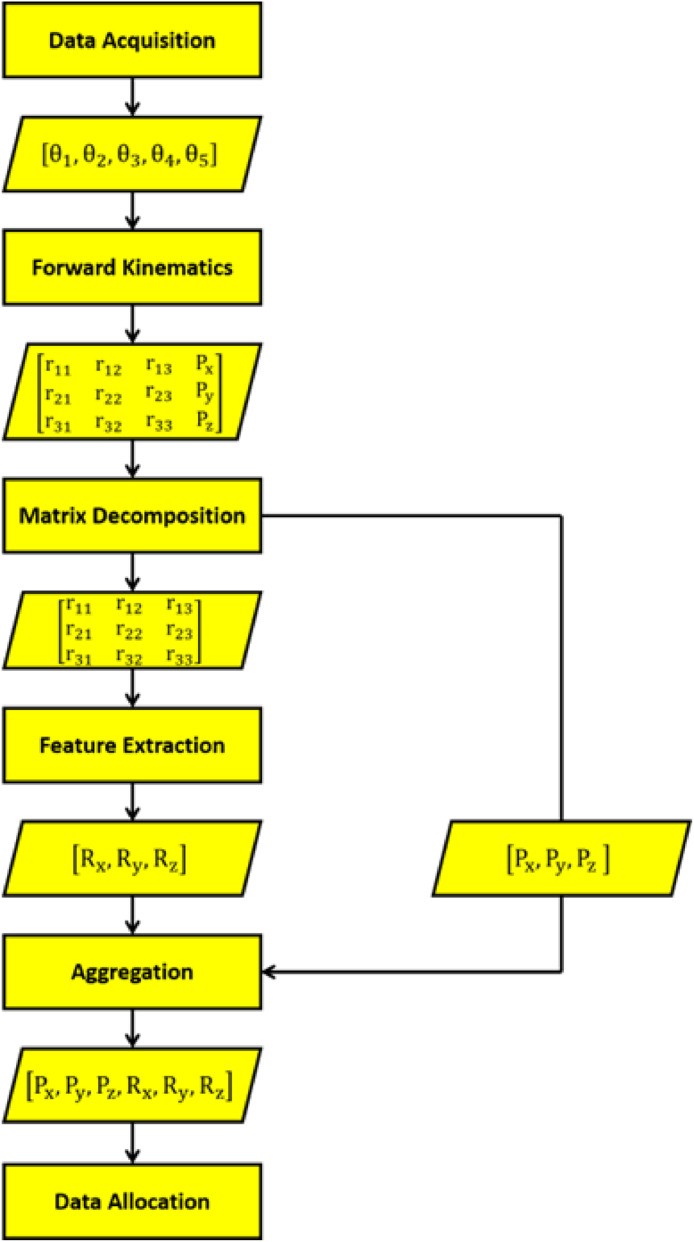
Experimental design.

The dataset plays a pivotal role in the implementation of artificial intelligence technologies, one of which is neural networks. The presence of this dataset is very important to build neural networks. Due to its important role, we are motivated to create the dataset to develop the neural networks-based inverse kinematics model for NAO robot arms. This dataset can be used as a means of establishing the robotic motion control for NAO robot arms. The experimental design to generate this dataset can be illustrated in Fig. 3. The sequential steps to generate this dataset are as follows: (1) data acquisition, (2) modeling the forward kinematics of NAO robot arms, (3) matrix decomposition, (4) feature extraction, (5) aggregation, and (6) data allocation.

## 5 Materials

### 5.1 Data acquisition

The NAO robot is physically similar to the human body structure because of containing a head, two arms, and two legs. This humanoid robot is available in two models: NAO H21 and NAO H25. Compared with the NAO H21, the NAO H25 has more degrees of freedom (DOF). In total, the NAO H21 has 21 DOF, while the NAO H25 has 25 DOF distributed on its head, two arms, and two legs. The main difference between these two models lies in the number of joints and the type of end-effector on their arms. Meanwhile, for the other parts of the body, the number of joints and the type of end-effector are the same. For this reason, our discussion will be more focused on the robotic arms of the NAO H25. The NAO robot version that we used for the data acquisition is the NAO H25 v3.3.

**Fig. 4:**
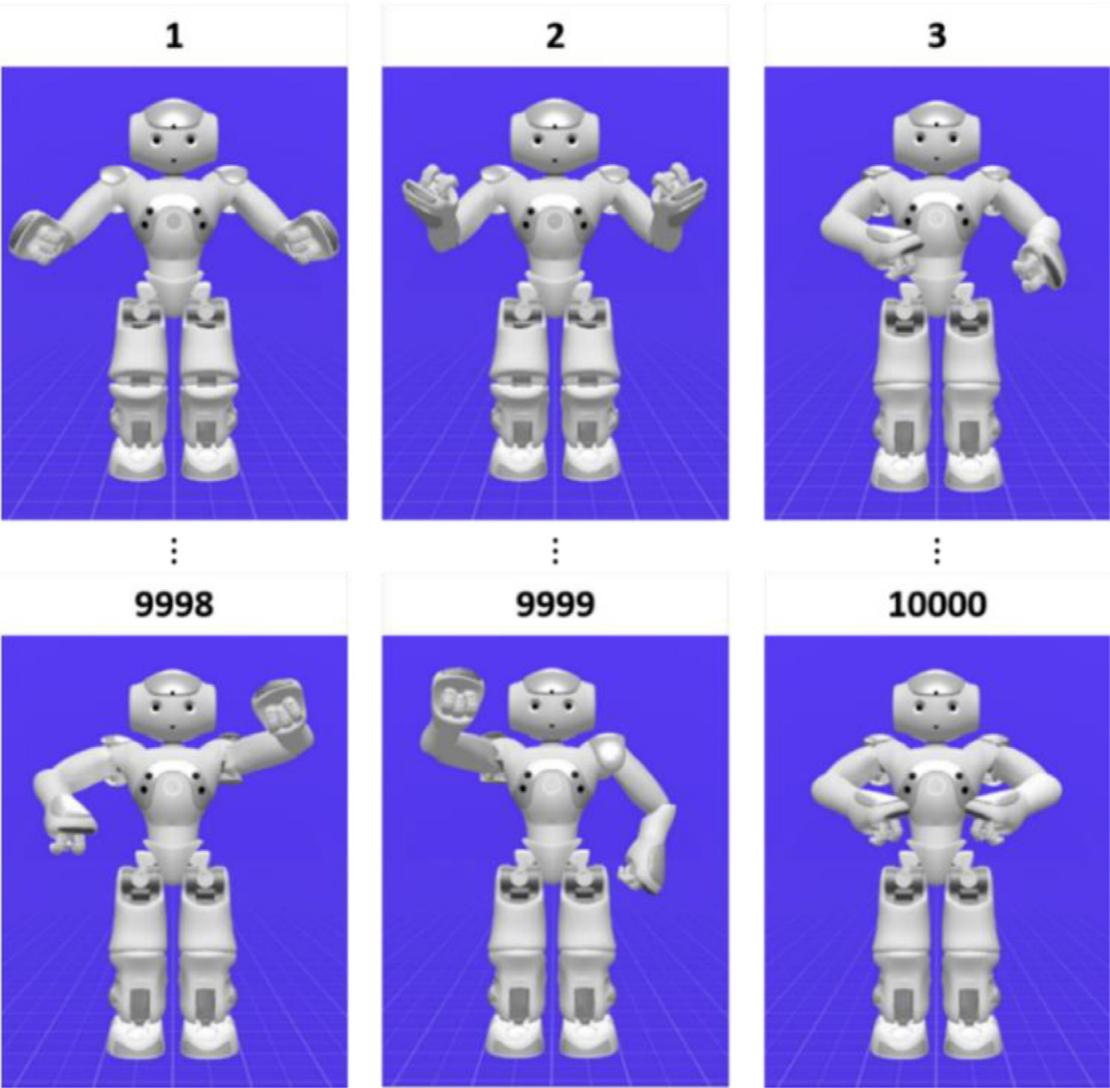
The data sample of the NAO robot arm poses.

The NAO robot arms kinematically contain a series of rigid links connected by revolute joints. The assembly of rigid links connected by the revolute joints forms the kinematic chain [10]. The part located in the last kinematic chain is a so-called end-effector. For each arm, the NAO H25 has five revolute joints and a prehensile hand as the end-effector [9]. The movement direction of the end-effector in the workspace is driven by revolute joints, each of which can move independently with respect to others. By varying the angular positions of these revolute joints, we can create various robotic arm poses. In the data acquisition, we generated 10000 robotic arm poses by using choregraphe software. Fig. 4 shows the data sample for the robotic arm poses. To avoid data redundancy, these robotic arm poses should be unique to one another.

### 5.2 A set of joint angular positions

From the data acquisition, we obtained 10000 robotic arm poses. As previously mentioned, the robotic arm poses are created from various joint configurations. Therefore, by extracting the robotic arm poses, we can obtain the data containing a set of joint angular positions of the NAO robot arms. The data samples for the joint angular positions are presented in Table 3 and Table 4, respectively. These tables have fields (columns) and records (rows). The data fields represent the joints involved in a robotic arm, while the data records are a set of joint angular positions. The joint angular positions that we provided in this dataset are in the radian unit.

**Table 3:** A set of joint angular positions extracted from the NAO's left arm poses.

No	$\theta_{1}$ (rad)	$\theta_{2}$ (rad)	$\theta_{3}$ (rad)	$\theta_{4}$ (rad)	$\theta_{5}$ (rad)
1	1.45139	0.81636	0.43327	-0.86791	-0.47177
2	0.65871	1.17880	-0.68507	-0.54854	-0.53581
3	0.15589	0.60224	-0.14855	-0.96952	-0.42192
4	-0.96937	0.45809	-0.17042	-0.74558	-0.00880
5	-0.66673	0.32475	0.30164	-1.10651	0.73428
⋮	⋮	⋮	⋮	⋮	⋮
9996	0.97719	0.56258	-1.70654	-1.00938	-0.71481
9997	0.83020	0.51138	-1.63072	-1.05069	-0.81798
9998	0.68320	0.46018	-1.55489	-1.09200	-0.92114
9999	0.53621	0.40899	-1.47907	-1.13330	-1.02431
10000	0.38921	0.35779	-1.40324	-1.17461	-1.12748

**Table 4:** A set of joint angular positions extracted from the NAO's right arm poses.

No	$\theta_{1}$ (rad)	$\theta_{2}$ (rad)	$\theta_{3}$ (rad)	$\theta_{4}$ (rad)	$\theta_{5}$ (rad)
1	-0.98727	-1.03049	-0.29515	1.19638	0.01696
2	0.49338	-0.92155	-1.00017	0.70232	1.33316
3	-1.32138	-0.60855	0.25970	0.48005	-0.29894
4	0.85806	-0.32185	-0.41366	0.38794	-0.00819
5	0.46961	-0.66923	0.44363	0.88413	-0.74223
⋮	⋮	⋮	⋮	⋮	⋮
9996	-1.72477	-0.86733	0.21370	1.18898	-1.45081
9997	1.20772	-0.77787	0.35601	0.90353	-0.34025
9998	1.26974	-0.81317	-0.30928	1.23261	-0.19054
9999	-1.13431	-0.96883	0.14071	0.84755	-0.65852
10000	1.98000	-0.98339	-0.11957	0.31039	-0.28480

## 6 Methods

### 6.1 Forward kinematics

Forward kinematics is concerned with the mapping from the joint space to the end-effector operational space, which is the cartesian space. The forward kinematics is also referred to as direct kinematics. The forward kinematics has a specific function to calculate the end-effector position and orientation in the cartesian space based on a given set of joint angular positions. Because of its important role, the forward kinematics must be formulated correctly. The way to formulate the forward kinematics of the NAO robot arms has been entirely explained in our previous research [1]. In short, the forward kinematics can be obtained by multiplying a set of the transformation matrices, each of which is the result of transformation between two adjacent coordinate frames from the torso as the base coordinate frame to the hand as the end-effector. The forward kinematics is typically defined in a homogeneous transformation matrix, as shown in Eq. (1).

(1) $$T_{Hand}^{Torso}=T_{0}^{Torso}\;T_{1}^{0}\left(\theta_{1}\right)\;T_{2}^{1}\left(\theta_{2}\right)\;T_{3}^{2}\left(\theta_{3}\right)\;T_{4}^{3}\left(\theta_{4}\right)\;T_{5}^{4}\left(\theta_{5}\right)\;T_{Hand}^{5}$$

$$T_{Hand}^{Torso}=\left[\begin{array}{cccc} r_{11} & r_{12} & r_{13} & P_{x}\\ r_{21} & r_{22} & r_{23} & P_{y}\\ r_{31} & r_{32} & r_{33} & P_{z}\\ 0 & 0 & 0 & 1 \end{array}\;\right]$$

where {[$r_{11},r_{12},r_{13}$], [$\;r_{21},r_{22},r_{23}$], [$\;r_{31},r_{32},r_{33}$]} represents the end-effector orientation and [$P_{x},P_{y},P_{z}$] denotes the end-effector position.

### 6.2 Matrix decomposition

As mentioned in Section 5.1, the result of the forward kinematics is generally expressed in a homogeneous transformation matrix. This transformation matrix consists of 12 main elements that denote the end-effector position and orientation in the cartesian space. This transformation matrix can be then decomposed into the 3×1 translation matrix and the 3×3 rotation matrix, as shown in Eqs. (2)–(3). The translation matrix describes the end-effector position with respect to the torso, while the rotation matrix indicates the end-effector orientation relative to the torso's coordinate frame.

(2) $$P_{Hand}^{Torso}=\left[\begin{array}{c} P_{x}\\ P_{y}\\ P_{z} \end{array}\right]$$

(3) $$R_{Hand}^{Torso}=\left[\begin{array}{ccc} r_{11} & r_{12} & r_{13}\\ r_{21} & r_{22} & r_{23}\\ r_{31} & r_{32} & r_{33} \end{array}\right]$$

### 6.3 Feature extraction

From the matrix decomposition, we obtained the translation matrix and rotation matrix. The translation matrix has three elements, each of which denotes the end-effector position in the three-dimensional cartesian space. Meanwhile, the rotation matrix contains nine elements. However, only three elements of them are independent [11]. It indicates that the rotation matrix gives a redundant description for the end-effector orientation in the three-dimensional cartesian space. For this reason, we require feature extraction. The objective of the feature extraction is to reduce the number of features in the data by means of creating new features from the existing ones and then followed by eliminating unnecessary features. In this case, the feature extraction will be carried out by transforming the rotation matrix to rotation vectors. The way to transform the rotation matrix to rotation vectors can be conducted by the following equations [12,13]

(4) $$\phi =arcos\left(\frac{r_{11}+r_{22}+r_{33}-1}{2}\right)$$

(5) $$\left[\begin{array}{c} a_{x}\\ a_{y}\\ a_{z} \end{array}\right]=\frac{1}{2\sin\phi }\left[\begin{array}{c} r_{32}-r_{23}\\ r_{13}-r_{31}\\ r_{21}-r_{12} \end{array}\right]$$

(6) $$\left[\begin{array}{c} R_{x}\\ R_{y}\\ R_{z} \end{array}\right]=\phi \left[\begin{array}{c} a_{x}\\ a_{y}\\ a_{z} \end{array}\right]$$

Alternatively, to make the transformation from the rotation matrix to the rotation vectors easier, we can use SciPy library [14]. In general, the end-effector orientation represented in the rotation matrix contains nine parameters. The drawback of the rotation matrix representation is that there are so many parameters, which makes it difficult to interpret. As a result of the feature extraction, the end-effector orientation can be represented by only using three parameters.

### 6.4 Aggregation

The end-effector position in the three-dimensional cartesian space is denoted by using three parameters [$P_{x},P_{y},P_{z}$], whereas the end-effector orientation in the three-dimensional cartesian space is also denoted by using three parameters [$R_{x},R_{y},R_{z}$]. Accordingly, the total number of the parameters that represent the end-effector position and orientation in the three-dimensional cartesian space is six parameters.

(7) $$T_{Hand}^{Torso}=\left[\begin{array}{c} \begin{array}{c} P_{x}\\ P_{y} \end{array}\\ \begin{array}{c} P_{z}\\ R_{x} \end{array}\\ \begin{array}{c} R_{y}\\ R_{z} \end{array} \end{array}\right]$$

## 7 Experimental Result

### 7.1 The end-effector position and orientation

The experiment has been conducted to create the dataset. From the data acquisition that we explained in the previous section, we obtained data composed of a set of joint angular positions. Besides, we also require the end-effector position and orientation data. By using the forward kinematics, we can generate the data containing the end-effector position and orientation. It can be conducted by feeding a set of joint angular positions into the forward kinematics Eq. (1). Because of containing a redundant description for the end-effector orientation, we decided to conduct feature extraction. In this case, the feature extraction aims to reduce the redundancy and to give a simpler representation for the end-effector orientation. The feature extraction was conducted by transforming the rotation matrix to rotation vectors using Eqs. (4-6). In this way, the end-effector position and orientation can be represented by only using six parameters, each of which is independent. From the experimental result, we acquired the data comprising the end-effector position and orientation. The data samples for the end-effector position and orientation of the NAO robot arms can be seen in Tables 5 and 6.

**Table 5:** The end-effector position and orientation of the NAO's left arm.

No	$P_{x}\;\left(mm\right)$	$P_{y}\left(mm\right)$	$P_{z}\left(mm\right)$	$R_{x}\left({}^{\circ }\right)$	$R_{y}\left({}^{\circ }\right)$	$R_{z}\left({}^{\circ }\right)$
1	-27.92	182.21	-69.34	-7.65	101.56	8.92
2	110.58	265.71	55.77	-42.64	-5.20	41.60
3	180.96	123.82	74.79	-31.37	7.07	-19.69
4	110.66	124.68	262.95	0.00	-60.22	-19.13
5	164.02	78.23	180.94	67.21	-38.17	-28.21
⋮	⋮	⋮	⋮	⋮	⋮	⋮
9996	152.92	199.05	53.72	-105.85	-27.21	7.23
9997	162.58	184.36	75.48	-112.66	-27.85	-5.82
9998	167.76	169.30	99.22	-118.65	-26.87	-19.84
9999	168.08	154.01	123.90	-122.13	-24.76	-34.41
10000	163.37	138.66	148.40	-124.24	-21.14	-49.18

**Table 6:** The end-effector position and orientation of the NAO's right arm.

No	$P_{x}\;\left(mm\right)$	$P_{y}\left(mm\right)$	$P_{z}\left(mm\right)$	$R_{x}\left({}^{\circ }\right)$	$R_{y}\left({}^{\circ }\right)$	$R_{z}\left({}^{\circ }\right)$
1	116.12	-180.97	198.59	-10.39	-41.23	5.04
2	83.41	-228.19	-25.54	24.58	52.19	-43.12
3	45.81	-187.38	285.07	2.08	-81.96	-9.99
4	112.87	-146.16	-74.21	-19.96	56.73	13.66
5	175.85	-162.01	41.35	-21.91	5.63	10.07
⋮	⋮	⋮	⋮	⋮	⋮	⋮
9996	-45.30	-164.71	261.67	-64.67	-103.05	-62.57
9997	80.83	-173.19	-58.22	-2.33	52.92	6.99
9998	5.41	-146.41	-65.55	-1.36	83.66	35.27
9999	65.97	-213.95	245.25	-24.23	-66.84	-26.58
10000	-66.36	-268.20	-14.12	-52.20	116.57	-3.48

### 7.2 Data allocation

**Fig. 5:**
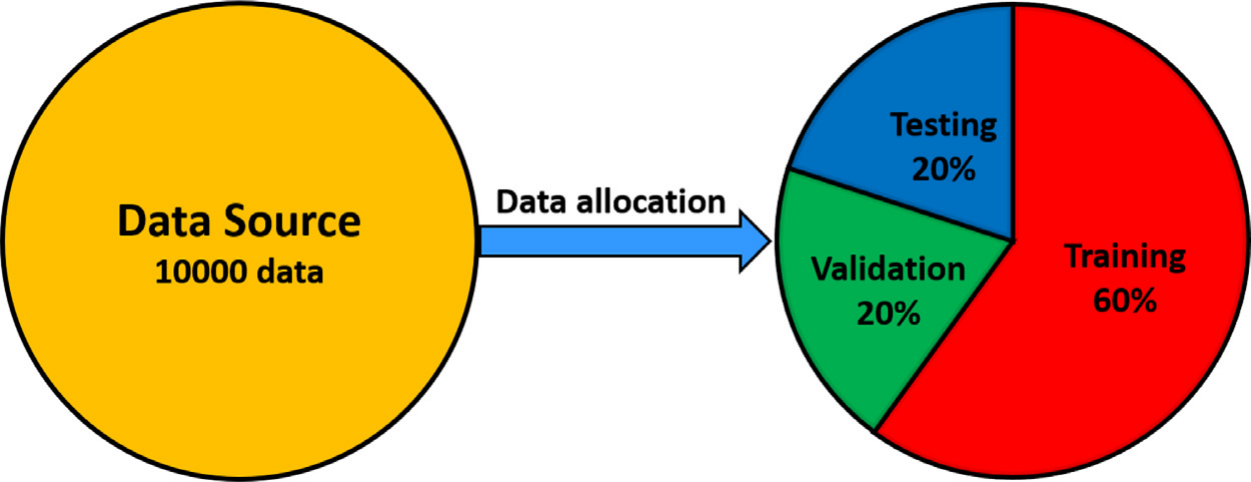
Data allocation.

The dataset to build neural networks-based inverse kinematics model for NAO robot arms consists of 10000 input-output data pairs, where the input data is the end-effector position and orientation and the output data is a set of joint angular positions. This dataset should be then split into three partitions: the training dataset, validation dataset, and testing dataset [6]. Each of them has a different function. The training dataset is used for training the neural networks. The validation dataset is then applied to validate the neural networks’ performance during the training process. Moreover, the validation dataset can be also used to tune hyperparameters of the neural networks. Meanwhile, the testing dataset is employed post-training to test how well the performance of the trained neural networks. As depicted in Fig. 5, from 10000 data, 60 % of data was allocated for the training dataset, 20 % of data for the validation dataset, and the other 20 % of data for the testing dataset.

**Fig. 6:**
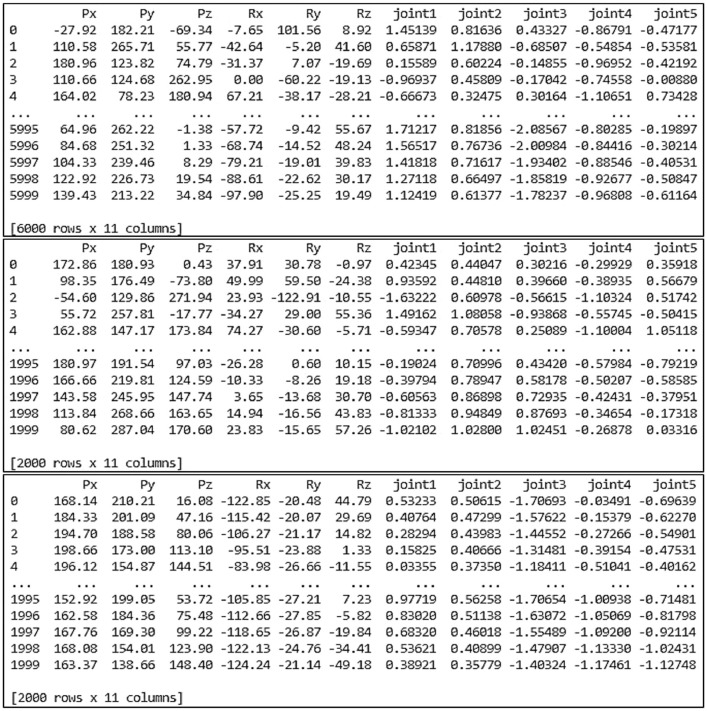
Data allocation for the NAO's left arm.

Fig. 6 presents the result of data allocation for the NAO's left arm. This dataset contains the input-output data pairs. For the input data, there are six input parameters. In the input parameters, the end-effector position is denoted by the position vectors [$P_{x},P_{y},P_{z}$], and the end-effector orientation is represented by the rotation vectors [$R_{x},R_{y},R_{z}$]. On the other hand, there are five output parameters for the output data. These output parameters are the left shoulder pitch joint ($\theta_{1}$), left shoulder roll joint ($\theta_{2}$), left elbow yaw joint ($\theta_{3}$), left elbow roll joint ($\theta_{4}$), and left wrist yaw joint ($\theta_{5}$), respectively. As a result of data allocation, 6000 data was provided for the training dataset, 2000 data for the validation dataset, and the rest of 2000 data for the testing dataset.

**Fig. 7:**
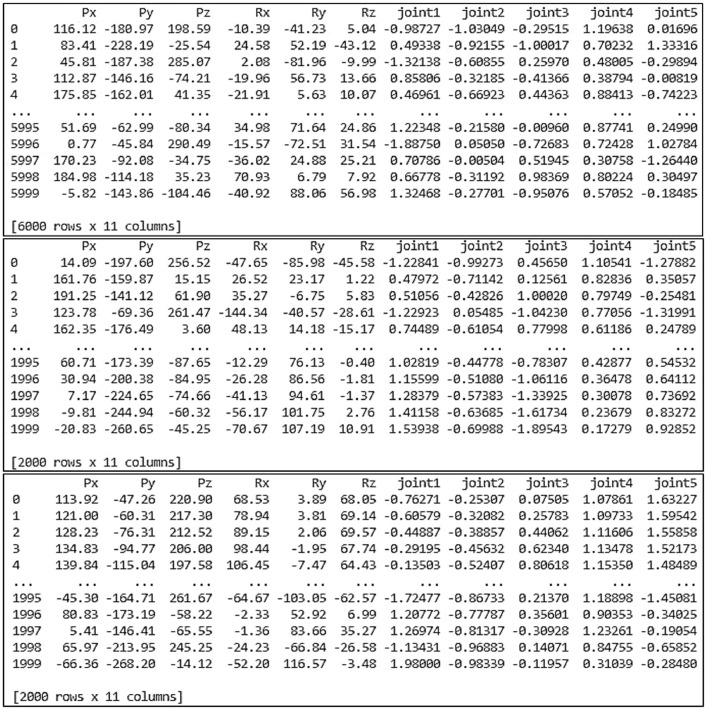
Data allocation for the NAO's right arm.

Meanwhile, the result of data allocation for the NAO's right arm can be seen in Fig. 7. This dataset is composed of input-output data pairs. There are six input parameters for the input data. In the input parameters, the end-effector position is denoted by the position vectors [$P_{x},P_{y},P_{z}$], while the end-effector orientation is expressed by the rotation vectors [$R_{x},R_{y},R_{z}$]. On the other side, there are five output parameters for the output data. These output parameters include the right shoulder pitch joint ($\theta_{1}$), right shoulder roll joint ($\theta_{2}$), right elbow yaw joint ($\theta_{3}$), right elbow roll joint ($\theta_{4}$), and right wrist yaw joint ($\theta_{5}$). From the data allocation, 6000 data can be used for the training dataset, 2000 data for the validation dataset, and the remaining 2000 data for the testing dataset.

**Fig. 8:**
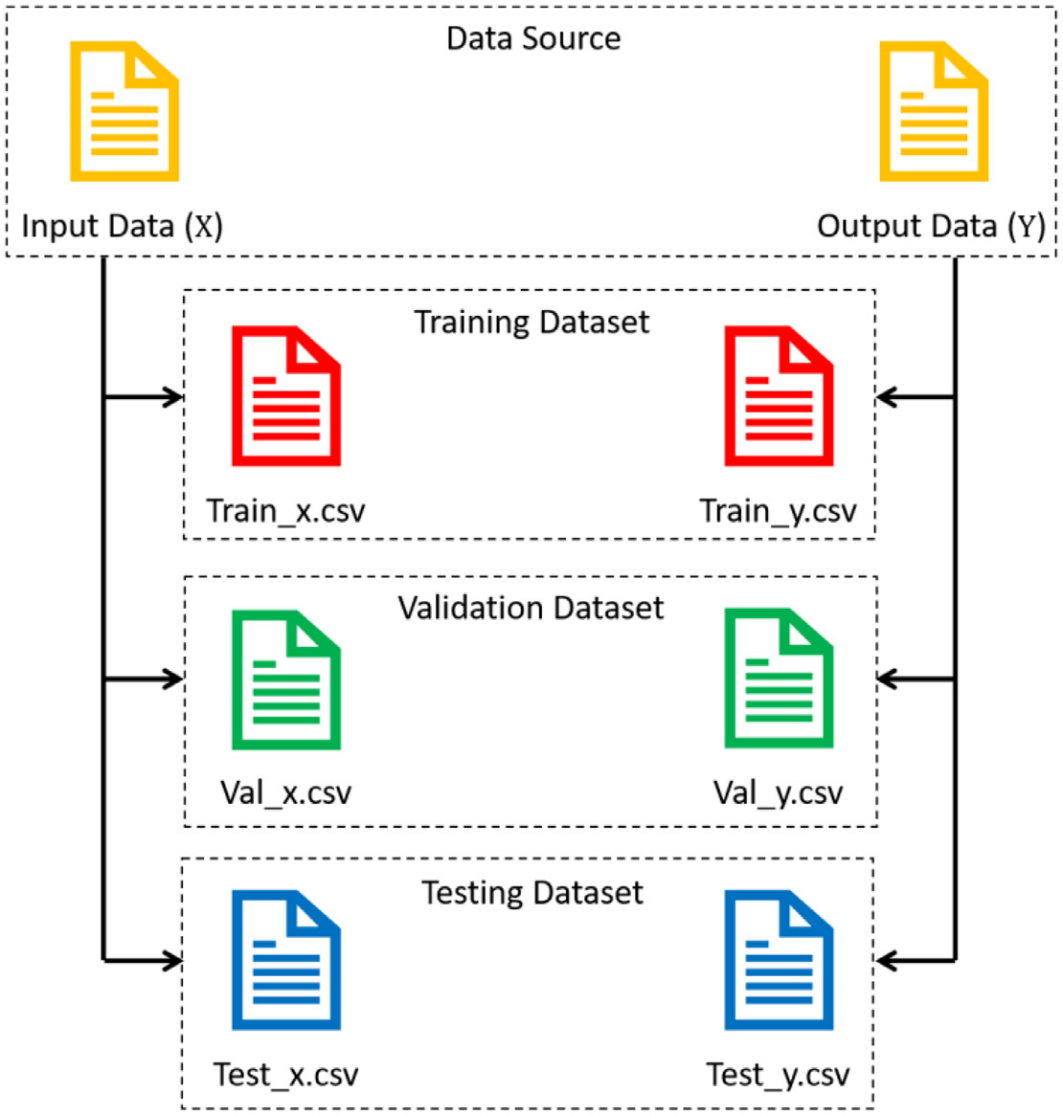
CSV files in the dataset.

The dataset that we generated in this research is entirely in CSV format. CSV stands for Comma Separated Values. This file format has been widely used for saving data. A CSV file is generally tabular data that is saved as plain text. This file uses commas to separate values in tabular data. It should be noted that the kind of dataset in this paper is tabular data. As mentioned earlier, this dataset contains the input-output data pairs. For further applications, this dataset is divided into three partitions: training dataset, validation dataset, and testing dataset. This dataset is entirely stored in CSV format whose file names can be seen in Fig. 8. The files saved in CSV format have a .csv extension. One of the main advantages of storing data in CSV format is that the CSV file can be opened and viewed in any program application, such as Notepad, Microsoft Excel, Google Spreadsheet, and so on. Moreover, because the CSV file is plain text, it requires less storage capacity than the other file formats.

This dataset consists of input and output data pairs, so this dataset is specifically addressed on supervised learning. Supervised learning is such a paradigm where the neural networks are trained by a set of labeled data. Labeled data means that each input has its corresponding output or desired target. In supervised learning, the objective is to find the relationship model between the inputs and outputs. In this dataset, the inputs are the end-effector position and orientation in three-dimensional cartesian space, and the outputs are a set of joint angular positions in the joint space. The relationship model resulted from this learning is called the neural networks-based inverse kinematics model. The number of data provided in this paper is sufficient to build the neural networks-based inverse kinematics model for NAO robot arms. In supervised learning, where the dataset contains input and output data pairs, the number of required data is typically not as many as in unsupervised learning. This is because the labeled data in supervised learning provides guidance in the learning process. This guidance reduces the ambiguity in the learning process, thereby making it possible to achieve good performance with a smaller number of data compared to unsupervised learning. Different from supervised learning, unsupervised learning typically requires more data to achieve good results because it has to learn from unlabeled data.

## Limitations

The dataset that we provided in this paper is compatible with the NAO H25 v3.3 or later. However, this dataset is incompatible with the previous version.

## Ethics Statement

The authors declare that our dataset meets the ethical requirements for publication in Data in Brief and our work does not involve human subjects, animal experiments, or any data collected from social media platforms.

## CRediT authorship contribution statement

**Arif Nugroho:** Conceptualization, Methodology, Formal analysis, Visualization, Data curation, Software, Writing – original draft, Writing – review & editing. **Eko Mulyanto Yuniarno:** Methodology, Formal analysis, Validation, Investigation, Writing – review & editing. **Mauridhi Hery Purnomo:** Conceptualization, Supervision, Validation, Writing – review & editing.

## Data Availability

ARKOMA: The Dataset to Build Neural Networks-Based Inverse Kinematics for NAO Robot Arms (Original data) (Mendeley Data).
